# Evidence for cerebral edema, cerebral perfusion, and intracranial pressure elevations in acute mountain sickness

**DOI:** 10.1002/brb3.437

**Published:** 2016-02-05

**Authors:** Dana M. DiPasquale, Stephen R. Muza, Andrea M. Gunn, Zhi Li, Quan Zhang, N. Stuart Harris, Gary E. Strangman

**Affiliations:** ^1^ Psychiatry DepartmentMassachusetts General HospitalHarvard Medical SchoolCharlestownMassachusetts; ^2^ Environmental Medicine and Military Performance DivisionU.S. Army Research Institute of Environmental MedicineNatickMassachusetts; ^3^ Center for Space MedicineBaylor College of MedicineHoustonTexas; ^4^ Department of Emergency MedicineDivision of Wilderness MedicineMassachusetts General HospitalHarvard Medical SchoolBostonMassachusetts

**Keywords:** Altitude, exercise, hemoglobin, hypobaria, hypoxia, illness, near‐infrared spectroscopy, normobaric, optic nerve sheath

## Abstract

**Introduction:**

We hypothesized that cerebral alterations in edema, perfusion, and/or intracranial pressure (ICP) are related to the development of acute mountain sickness (AMS).

**Methods:**

To vary AMS, we manipulated ambient oxygen, barometric pressure, and exercise duration. Thirty‐six subjects were tested before, during and after 8 h exposures in (1) normobaric normoxia (NN; 300 m elevation equivalent); (2) normobaric hypoxia (NH; 4400 m equivalent); and (3) hypobaric hypoxia (HH; 4400 m equivalent). After a passive 15 min ascent, each subject participated in either 10 or 60 min of cycling exercise at 50% of heart rate reserve. We measured tissue absorption and scattering via radio‐frequency near‐infrared spectroscopy (NIRS), optic nerve sheath diameter (ONSD) via ultrasound, and AMS symptoms before, during, and after environmental exposures.

**Results:**

We observed significant increases in NIRS tissue scattering of 0.35 ± 0.11 cm^−1^ (*P* = 0.001) in subjects with AMS (i.e., AMS+), consistent with mildly increased cerebral edema. We also noted a small, but significant increase in total hemoglobin concentrations with AMS+, 3.2 ± 0.8 *μ*molL^−1^ (*P* < 0.0005), consistent with increased cerebral perfusion. No effect of exercise duration was found, nor did we detect differences between NH and HH. ONSD assays documented a small but significant increase in ONSD (0.11 ± 0.02 mm; *P* < 0.0005) with AMS+, suggesting mildly elevated ICP, as well as further increased ONSD with longer exercise duration (*P* = 0.005).

**Conclusion:**

In AMS+, we found evidence of cerebral edema, elevated cerebral perfusion, and elevated ICP. The observed changes were small but consistent with the reversible nature of AMS.

## Introduction

The underlying pathophysiology of acute mountain sickness (AMS) remains poorly understood. It has long been hypothesized that AMS is related to cerebral edema and/or elevations in intracranial pressure (ICP) (Sutton and Lassen 1979). Some evidence further suggests that AMS may be caused by mild cerebral edema of vasogenic origin (Hackett et al. 1998; Bailey et al. 2004; Wilson and Milledge 2008). Alternatively, it has been suggested that vasogenic edema is an adaptive response to hypoxic exposure and that AMS instead represents a maladaptive cytotoxic edema state (Fischer et al. 2004; Kallenberg et al. 2007; Lawley et al. 2013). More limited evidence suggests that not only hypoxia, but the hypobaria of high altitude may contribute to the development of AMS (Roach et al. 1996; Schommer et al. 2010; Fulco et al. 2011; DiPasquale et al. 2015), possibly through alterations in body fluid regulation (Roach et al. 1996), although this remains controversial (Girard et al. 2012; Millet et al. 2012a,b; Mounier and Brugniaux 2012). In addition, an association between exercise and increased AMS has been described (Roach et al. 2000; Mairer et al. 2012), but this has not been reliably found (Rupp et al. 2013). The relationship between AMS, exercise duration, and cerebral edema has been difficult to investigate due to logistical challenges of conducting brain imaging in hypoxic environments along with exercise.

The few studies using conventional MRI and CT scans to investigate cerebral changes with AMS have found inconsistent evidence of increased cerebral edema or swelling (Levine et al. 1989; Morocz et al. 2001; Mairer et al. 2012). Subjects in these prior studies could not be continuously exposed to altitude, however, requiring either descent from hypobaric hypoxia for scanning, or switching to normobaric hypoxia. Symptom reduction or the lack of sustained hypobaria may have contributed to the inconsistencies. It is thus preferred that cerebral monitoring be conducted before, during, and after ascent to high altitude while the key environmental conditions are sustained and the AMS pathophysiologic process is active.

Near‐infrared spectroscopy (NIRS) of the brain is a technique that can address this issue. Tissue is sufficiently transparent to near‐infrared wavelengths (650‐950 nm) to enable noninvasive monitoring of brain oxygenation and blood volume (Jobsis 1977; Strangman et al. 2002a,b; Huppert et al. 2006). Near‐infrared spectroscopy is sensitive to blood volume changes related to hemorrhage (Gopinath et al. 1993; Stankovic et al. 1999; Al‐Rawi and Kirkpatrick 2006), blood oxygenation changes related to ischemia or hypoxia, (Gopinath et al. 1995; Robertson et al. 1995; Zhang et al. 2000; Al‐Rawi and Kirkpatrick 2006) and the small oxygenation changes associated with brain activity (Strangman et al. 2002b; Huppert et al. 2006). With suitable instrumentation, one can also detect changes in cerebral fluids, as occurs in edema states, via light scattering (Villringer and Chance 1997). Near‐infrared spectroscopy detection sensitivity decreases with depth, with typical instruments being most sensitive to the outermost 1–2 cm of brain tissue (Strangman et al. 2013, 2014). Thus, while NIRS provides limited information about deep brain structures, global effects or regional changes near the surface of the brain are easily detected. Importantly, NIRS devices are compatible with environmental chambers where simulated altitude environments can be carefully controlled.

Hypothesized cerebral effects of AMS include edema, cerebral blood volume (CBV) changes, and/or oxygenation changes. Edema has been shown to alter tissue photon scattering in mice (Thiagarajah et al. 2005), and more recently, Xie and colleagues have showed that cerebral edema – in their case caused by traumatic brain injury (TBI) – increases photon scattering in the rat brain (Xie et al. 2011). Xie et al. (2011) reported brain water content (BWC) – determined by the desiccation method – was highly linearly related (*R* ^2^ = 0.87) to the scattering coefficient of near‐infrared light, *μ*_s_′. While similar validation has not been conducted in adult humans, numerous simulation and experimental studies have consistently demonstrated that thicker scalp and skull layers (e.g., from adult humans vs. smaller animals) only lead to reductions in sensitivity (Strangman et al. 2013, 2014) rather than any qualitative differences in the nature of the measurement. Thus, we hypothesized that NIRS could be used to identify changes in cerebral edema and perfusion during the development of AMS.

Examining the optic nerve sheath can provide useful, complementary data about cerebral status. Increases in ICP are transmitted by the cerebrospinal fluid down the perineural subarachnoid space of the optic nerve, expanding the optic nerve sheath diameter (ONSD). This can be measured by ultrasound imaging. Several studies have demonstrated that imaging and quantifying the optic nerve sheath diameter can provide a noninvasive measurement related to ICP (Geeraerts et al. 2008; Kimberly et al. 2008), although this can be variable (Hansen and Helmke 1997) and hence, provides more of a relative change rather than an absolute change in ICP. Recent studies have further demonstrated a clear relationship between ONSD and AMS in remote settings (Fagenholz et al. 2009), which implicates intracranial alterations in the development of AMS. What remains to be understood, however, are the relationships between cerebral edema, cerebral perfusion, ICP, and the development of AMS. Even less is known about the (patho)physiological brain changes and relative roles of exercise duration and hypobaria in the evolution of AMS symptoms.

In this study, we investigated the hypothesis that AMS is related to alterations in cerebral edema, perfusion, and ICP. We simultaneously investigated the role of hypobaria and exercise in the evolution of cerebral physiology in relation to AMS. To achieve this, we utilized radio‐frequency near‐infrared spectroscopy (RF‐NIRS) and ONSD ultrasonography techniques to measure cerebral parameters before, during, and after exposure to reduced barometric pressure, hypoxia, and exercise.

## Materials and Methods

### Subjects

Healthy subjects (*n* = 36; Table 1) participated in this study approved by the Institutional Review Boards of the Massachusetts General Hospital, the US Army Research Institute of Environmental Medicine and the Human Research Protection Office, US Army Medical Research and Materiel Command, and in compliance with the Code of Ethics of the World Medical Association (Declaration of Helsinki). Subjects were regular exercisers (at least 20 min, 3 day/week) who were born at altitudes less than 2134 m, living in areas that were less than 1220 m, and had not traveled to areas that were more than 1220 m for more than 2 days within the last 2 months. All subjects met Army height and weight standards. After providing verbal and written consent, subjects were medically cleared to participate via a clinical exam and routine blood and urine testing. We initially screened 195 volunteers, enrolled 39, and screened out an additional three during physical examination. One subject was unable to complete three of the subtests during one exposure due to illness.

**Table 1:** Study participant characteristics (mean ± SD). There were no significant differences among treatment groups (*P* > 0.05)

Age (year)	27.7 ± 7.8
Females	18/36
Height (cm)	171.4 ± 8.4
Weight (kg)	68.9 ± 10.3
BMI	23.4 ± 2.7
HR_rest_ (bpm)	63.7 ± 11.6

BMI, body mass index; SD, standard deviation.

### Overall design

To vary AMS severity, the study involved six experimental groups: three different exposure environments – normobaric normoxia (NN), normobaric hypoxia (NH), and hypobaric hypoxia (HH) crossed with two exercise durations: short exercise (10 min) and long exercise (60 min). Each subject participated in two different conditions out of the six, separated by at least 2 weeks, resulting in 72 total environmental exposures and a partial within‐/between‐subjects design.

### Testing protocol

**Figure 1:**
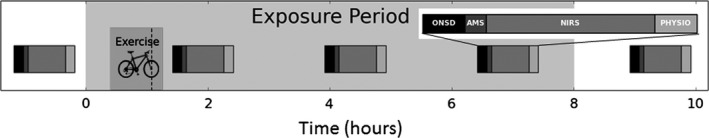
Schematic of experimental design. Testing periods occurred 5 times, before, during and after the 8 h exposure period, with all tests occurring in the same order each time (expanded inset). The dashed line indicates the start of the exercise period for short (10 min) exercise; both exercise periods ended at the same time.

Each subject participated in five visits. Visit 1 consisted of consent and medical screening. Visit 2 included baseline structural and functional MRI scanning. Visit 3 involved in‐laboratory training, device calibration, and pretesting for the two environmental exposure days. The timeline for each environmental exposure day (visits 4 and 5) appears in Figure 1. Subjects first performed 75 min of testing within the altitude chamber at sea level. They were then rapidly ascended (within 15 min) to their target environment and spent 8 h in that environment. During exposure, they performed their exercise condition followed by three 75 min testing sessions (separated by 2.5 h each). Subjects were tested a final time 1 h after return to sea level. All participants were advised to not exercise for more than 30 min (neither leg nor arm exercise) or consume alcohol in the 24 h preceding environmental exposure days. On environmental exposure days, regular coffee drinkers were permitted their usual morning beverage prior to testing, and all subjects were provided food and caffeine‐free drinks ad libitum for the remainder of the day.

### Environmental exposures

Subjects were naïve to the assigned conditions. They were not provided any information on which room was for NN, NH, or HH, and all research personnel used supplemental oxygen regardless of the condition. NN was performed in the hypobaric chamber at PB = 752 mmHg, which enabled secure sealing of the chamber door, further ensuring subject naivety (PIO_2_ = 147.3 mmHg; 300 m equivalent altitude). HH was performed in the hypobaric chamber (P_B_ = 439 mmHg; PIO_2_ = 81.9 mmHg; 4400 m equivalent altitude). NH was performed at ambient pressure in a hard vinyl‐sided hypoxia room (Colorado Altitude Training, Boulder, CO) with ambient oxygen partial pressure matched to the HH condition at 91.7 mmHg (P_B_ = 760 mmHg; PIO_2_ = 86.1 mmHg; 4400 m equivalent altitude). Following all testing, subjects were asked if they knew which conditions they participated in, and >90% could not or incorrectly guessed their experimental condition.

### Exercise

After ascent was complete, subjects assigned to 60 min of exercise began cycling immediately while subjects assigned to 10 min began 50 min later (dashed vertical line in Fig. 1), so that all exercise sessions ended at the same time of exposure. Cycling was performed at 52.1 ± 4.4% of heart rate reserve (HR_rsv_) (Excalibur Lode, Groningen, The Netherlands). HR_rsv_ was calculated using age‐predicted HR_max_ and HR_rest_ measured on a day prior to the environmental exposures to minimize anticipatory effects. Target HR was stabilized within 5–8 min. Absolute exercising workload was adjusted to maintain target HR. Exercising HR was measured via 3‐lead ECG (Physioflow, Poissy, France).

### Near‐infrared spectroscopy measurements

Near‐infrared spectroscopy measurements were made with a customized 4‐wavelength RF‐NIRS system (ISS, Champaign, IL), incorporating 690, 780, 830, and 850 nm light sources and two source‐detector separations (1.25 and 3.5 cm) in each of two sensor pads. For localization, structural (MEMPRAGE) and BOLD‐contrast functional MRI maps were imported into a Brainsight‐2 stereotactic system (Rogue Research, Montreal, Canada) and used to localize the two NIRS probes over regions of multitask response‐related activation in the anterior prefrontal cortex on a subject‐by‐subject basis. All locations were over the anterior middle frontal gyrus, typically within 1 cm of F3 and F4 in the International 10/20 system (Homan et al. 1987). Measurements were made at 12.5 Hz during quiet, seated rest to obtain stable baseline tissue perfusion and scattering measurements. Near‐infrared spectroscopy data was analyzed using custom software, portions of which are included in the HomER NIRS processing package. Additionally, the slope method (Hueber et al. 2001), which determines scattering (*μ*_s_'), oxy‐hemoglobin (HbO_2_), deoxyhemoglobin (HHb), and total hemoglobin (HbT) concentrations while simultaneously eliminating effects from overlying tissue layers such as scalp, was used. Since scattering affects all four wavelengths similarly, we computed a mean *μ*_s_' across wavelengths to reduce variability. Immediately after the resting baseline period, subjects conducted three 5 sec Valsalva maneuvers (at 40 sec intervals) followed by three 5 sec Mueller maneuvers (also at 40 sec intervals). Maneuvers were made by continuously exhaling (Valsalva) or inhaling (Mueller) against a fixed gradient to maintain a manometer pressure of 40 mmHg. Trials were dropped when this pressure could not be maintained (<1% of all trials). We then measured the initial hemodynamic response (baseline‐to‐peak/‐trough) associated with each maneuver. If ICP was substantially elevated, we hypothesized that the response to Valsalva (Mueller) maneuvers would be reduced (enhanced) due to cerebral counter pressure. For baseline measures, NIRS data was averaged over a 100 sec period during each recording session that was deemed free of motion artifacts (rare but occasionally occurred). For Valsalva (Mueller) maneuvers, we computed the change in HHb and HbO_2_ concentrations between the mean of the 5 sec prior to Valsalva (Mueller) and the response at the peak (trough).

### Optic nerve sheath diameter

Optic nerve sheath diameter (ONSD) measures were performed with a portable ultrasound system (Mindray, Mahwah, NJ) using a high‐frequency (7–10‐MHz) linear array transducer. All subjects were in a uniform, supine position. The probe was rested gently over the eye without applying pressure (thereby maintaining corneal convexity) and the optic nerve sheath was located. Three 5‐sec cines of horizontal section images through the optic nerve posterior to the orbit were collected on each eye during each of the five testing periods per exposure day. One image was obtained from each cine (three per eye) to minimize intraobserver variability (Ballantyne et al. 2002). For analysis, all cines were first stripped of any identifying or date information and randomized. From each 5‐sec recording, an optimal image was identified and saved. Then, as per previously reported procedures, the ONSD was measured 3 mm behind the retina of each eye (Fagenholz et al. 2009). Measurements were made by a single, highly trained technician, and cross validated by an independent expert for accuracy.

### Questionnaire

The Environmental Symptoms Questionnaire was administered to assess AMS. An AMS‐C score (Sampson et al. 1983) greater than or equal to 0.7 at a given time point indicated an individual has AMS (the AMS+ indicator variable).

### Statistical analysis

We fit linear mixed‐effects (multiple) regression models with cluster adjusted standard errors across subjects and experiment time points using Stata v11 software (StataCorp, LP, College Station, TX). Models using the AMS+ indicator variable as a predictor were used to identify those outcome variables associated with significant illness. Models with NH, HH, and long exercise indicator variables, plus time (in hours) as a continuous variable, were used to test for differences between NH and HH, and to identify changes associated with exercise and evolution over time. Chi‐squared analyses were used to directly compare NH and HH condition coefficients to test hypotheses related to hypobaria alone.

## Results

### Cerebral scattering outcome

**Figure 2:**
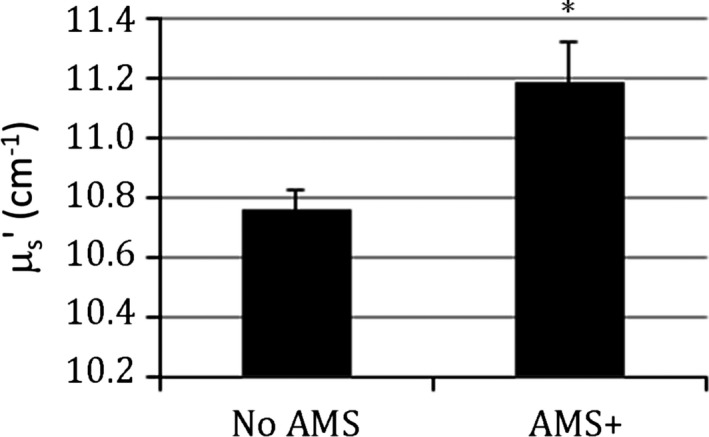
Difference in radio‐frequency near‐infrared spectroscopy scattering coefficient, *μ*_s_' (cm^−1^), for measurements on individuals with and without acute mountain sickness (AMS). Mean ± SE. *Significantly different than No AMS;* P* < 0.05.

Based on their AMS‐C scores, 46% of subjects developed AMS during hypoxia exposures (21% in NH, 67% in HH). Our primary hypothesis was that cerebral edema – as assessed via NIRS scattering – would be increased in individuals who had clinical AMS symptom levels relative to those who were not (Fig. 2). Linear mixed‐effects regression (Pinheiro and Bates 2000) on the mean scattering coefficient, *μ*_s_′, against AMS+ revealed a significant positive relationship wherein *μ*_s_′ was increased by 0.36 cm^−1^ (95% CI: 0.14–0.57 cm^−1^) in individuals with AMS (*Z*(*n* = 183) = 3.29, *P* = 0.001). The multiple linear regression fit including variables for NH, HH, exercise, and time is shown in Table 2, with significant increases in both NH and HH conditions but no significant effect of exercise duration or significant changes over time. There was not a significant difference between NH and HH, *χ* ^2^(1) = 0.69, *P* = 0.41.

**Table 2:** Regression results for RF‐NIRS scattering coefficient, *μ*_s_′, versus experimental conditions

Mean *μ*_s_′	Coefficient	SE	*z*	*P* > |*z*|	95% Confidence interval	
NH	0.353	0.1133	3.12	0.002	0.131	0.575
HH	0.261	0.1058	2.47	0.014	0.055	0.469
Hour	0.006	0.0126	0.48	0.634	−0.019	0.031
Long exercise	0.055	0.0883	0.62	0.533	−0.118	0.228
Intercept	10.59	0.1927	54.97	0.0001	10.22	10.97

RF‐NIRS, radio‐frequency near‐infrared spectroscopy; NH, normobaric hypoxia; HH, hypobaric hypoxia; SE, standard error.

### Hypoxia versus AMS

We added AMS+ as a regressor to the previous model (see Table 3) to test for the additive effects of hypoxia exposure and AMS on the NIRS scattering signal. The results suggest that having AMS is an additive effect over and above NH exposure, with a magnitude two‐thirds that of NH exposure alone. AMS was not an additive effect with respect to HH (*P* > 0.1), but this may have occurred because AMS+ was notably collinear with HH; that is, too many subjects in the HH condition became sick (67%) compared with NH subjects (25%). The collinearity was confirmed using condition diagnostics (Belsley et al. 1980). The effect of AMS+ was determined to be independent of NH and HH effects via a follow‐on model wherein the interactions of AMS+ with NH and HH were both nonsignificant (*P* > 0.4; not shown).

**Table 3:** Regression results for RF‐NIRS scattering coefficient, *μ*_s_′ (cm^−1^), versus experimental conditions and AMS

Mean *μ*_s_′	Coefficient	SE	*z*	*P* > |*z*|	95% Confidence interval	
NH	0.314	0.115	2.74	0.006	0.090	0.540
HH	0.176	0.113	1.55	0.120	−0.046	0.398
Long exercise	0.037	0.088	0.42	0.675	−0.136	0.201
AMS+	0.221	0.110	2.01	0.045	0.005	0.436
Intercept	10.63	0.186	57.14	0.0001	10.27	10.99

RF‐NIRS, radio‐frequency near‐infrared spectroscopy; AMS, acute mountain sickness; NH, normobaric hypoxia; HH, hypobaric hypoxia; SE, standard error.

### Cerebral perfusion and oxygenation

We also tested the hypothesis that AMS was associated with elevated total hemoglobin (HbT) concentrations (perfusion) and/or alterations in cerebral oxygenation. [HbT] was significantly elevated with AMS+, by 3.2 ± 0.8 *μ*molL^−1^, *Z*(*n* = 252) = 3.58, *P* < 0.0005. Using a mixed‐effects model for our experimental conditions (Table 4), again there were significant elevations in [HbT] for NH (3.82 *μ*molL^−1^) and HH (2.78 *μ*molL^−1^), and no significant changes over time or exercise were observed. The changes for NH and HH were again not significantly different from one another (*P* > 0.3).

**Table 4:** Regression results for RF‐NIRS derived total‐Hb concentration (Molar)

Mean [HbT]	Coefficient	SE	*z*	*P* > |*z*|	95% Confidence interval	
NH	3.82E‐06	1.06E‐06	3.62	0.0001	1.75E‐06	5.90E‐06
HH	2.78E‐06	9.89E‐07	2.81	0.005	8.40E‐07	4.72E‐06
Hour	5.63E‐08	1.61E‐07	0.35	0.727	−2.60E‐07	3.73E‐07
Long exercise	1.03E‐06	8.48E‐07	1.21	0.226	−6.36E‐07	2.69E‐06
Intercept	0.0000563	1.98e‐06	28.44	0.0001	0.0000524	0.0000602

RF‐NIRS, radio‐frequency near‐infrared spectroscopy; HbT, total hemoglobin; NH, normobaric hypoxia; HH, hypobaric hypoxia; SE, standard error.

**Figure 3:**
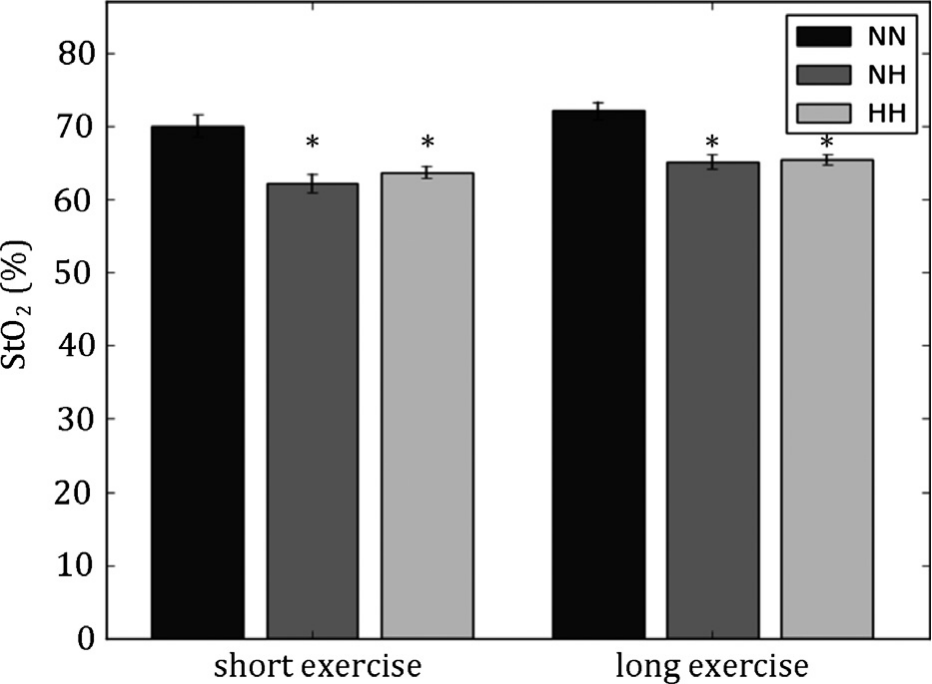
Tissue oxygenation (StO_2_) in the six experimental conditions. NH and HH conditions exhibited significantly lower StO_2_ than NN (*P < 0.05). NH and HH were not statistically different. Mean ± SE.

To confirm that our measurements sensitively reflected the hypoxic exposures, we calculated tissue oxy‐ and deoxyhemoglobin concentrations – and from this tissue oxygenation ([O_2_Hb]/([HHb]+[O_2_Hb]) – during the exposure period from only the close (1.25 cm) source‐detector pair, which measures scalp oxygenation (i.e., not using the slope analysis method). The results appear in Figure 3, revealing a tissue saturation of 71.1 ± 0.7% for controls during the exposure period, 63.6 ± 0.6% in NH and 64.7 ± 0.4% in HH, consistent with prior studies (Hueber et al. 2001). Both NH and HH were significantly different from controls (*P* < 0.0001), whereas NH and HH tissue oxygenations were not significantly different from one another (*P* > 0.2). When analyzing our NIRS data via the slope method, cerebral oxygenation was not found to be significantly altered by NH or HH (*P* > 0.1). While this would appear contrary to the results in Figure 3, it is important to remember that the slope method analysis implicitly corrects for common mode effects by canceling out changes (e.g., sensor contact, oxygenation, or other parameters) that are seen both in the near measurement (sensitive to superficial layers such as scalp) as well as in the far measurement (sensitive to deeper layers such as brain). This provides higher sensitivity to brain tissue, at the expense of masking system‐wide changes. The negative finding here thus implies that there was no additional change in oxygenation in NH or HH above and beyond the systemic effect observed in scalp tissue.

Given the nature of the RF‐NIRS technology, it is possible for “crosstalk” to occur between measurements, wherein a change in scattering is interpreted as a change in absorption (*μ*_a_) or vice versa. Since the identified changes in both scattering and absorption were small, we conducted two follow‐up titration experiments with our ISS instrument. In the first, a solution of India ink was diluted while maintaining fixed 1% Intralipid concentration (*μ*_s_′ = 10), measuring *μ*_a_ and *μ*_s_′ at six dilution steps. Regression analysis revealed a mean ∆*μ*_s_′ = 16.9 ± 2.7 cm^−1^ per unit change in *μ*_a_. Thus, the observed ∆[HbT] = +3.3 *μ*molL^−1^, equivalent to ∆*μ*_a_ = 0.005, would be expected to generate a crosstalk ∆*μ*_s_′ = 0.08 ± 0.01 cm^−1^. This is less than a quarter of the observed scattering change and hence not a substantial contributor to our scattering results. In the second titration, a 1% concentration of Intralipid was diluted with an India ink and water solution to maintain a constant absorption (*μ*_a_ = 0.0133) while decreasing scattering. We again measured *μ*_a_ and *μ*_s_′ at six dilution steps (up to a 30% reduction in *μ*_s_′). Regression analysis of *μ*_a_ versus *μ*_s_′ gave a mean (across wavelengths) change of 0.008 ± 0.002 in *μ*_a_ per unit change in *μ*_s_′. The observed Δ*μ*_s_′ = 0.35 cm^−1^ would be expected to generate crosstalk of ∆*μ*_a_ = 0.0028 ± 0.0007, or ∆[HbT] = 1.8 ± 0.4 *μ*molL^−1^. Thus, the potential crosstalk from the observed *μ*_s_′ change of 0.35 cm^−1^ to [HbT] was approximately half of the observed ∆[HbT] = 3.3 *μ*molL^−1^. The crosstalk error bounds cannot explain the entire observed increase in [HbT], but it is possible that crosstalk from our observed scattering change influenced our [HbT] concentration estimates.

### Valsalva/Mueller maneuvers

There was no significant modulation of the peak response with AMS+, NH, HH, or long exercise on either maneuver (*P* > 0.1 in all cases).

### Optic nerve sheath diameter

To address the question of whether ICP alterations may be associated with AMS and altitude, we first performed linear regression of the ONSD measures against AMS+, revealing a significant positive relationship wherein ONSD increased by 0.11 ± 0.02 mm in individuals with AMS+ (*Z*(*n* = 1719) = 4.2, *P* < 0.0005). When using a mixed‐effects multiple linear regression model with our experimental conditions, all effects were significant, including an effect of exposure time (Table 5). In this case, the estimated diameter change was smaller (0.059 mm for NH and 0.049 for HH), while long exercise provided an additional increase of 0.05 mm in ONSD diameter, and ONSD diameter also increased by 0.015 mm per hour of exposure. There was no significant difference in ONSD between NH and HH, *χ* ^2^(1) = 0.15, *P* = 0.70.

**Table 5:** Regression results for the optic nerve sheath diameter

Mean ONSD (mm)	Coefficient	SE	*z*	*P* > |*z*|	95% Confidence interval	
NH	0.0059	0.0026	2.24	0.025	0.0007	0.0111
HH	0.0049	0.0025	1.96	0.050	7.66E‐06	0.0098
Hour	0.0015	0.0003	4.91	0.0001	0.0009	0.0021
Long exercise	0.0057	0.0020	2.82	0.005	0.0017	0.0097
Intercept	0.4518	0.0045	99.33	0.0001	0.4429	0.4607

ONSD, optic nerve sheath diameter; NH, normobaric hypoxia; HH, hypobaric hypoxia; SE, standard error.

Prior reports have suggested body mass index (BMI) as a significant factor in AMS (McDevitt et al. 2014). Although our subjects had a fairly narrow range of BMI (see Table 1), as a check analysis we investigated whether including BMI in any of the above models qualitatively altered the results. BMI was not found to be significantly related to any of our outcome measures (*P* > 0.1).

## Discussion

In this study, we investigated three cerebral measures previously hypothesized to be related to the development of AMS, normobaric hypoxia, and hypobaric hypoxia during environmental exposures. The findings included: (1) a significant increase in light scattering through brain tissue, *μ*_s_′, previously associated with cerebral edema; (2) a significant but small increase in [HbT] associated with AMS+, suggesting mildly increased cerebral perfusion; and (3) a significant but small increase in ONSD associated with AMS illness, suggesting mildly elevated ICP.

Our first finding was the significant increase in scattering. Changes in light scattering in tissue can arise from various causes, specifically including alterations in refractive index, particle size distributions, or tissue density. Hemorrhage and edema are the most common cerebral changes that would alter these properties. However, hemorrhage would also be associated with a strong increase in light absorption and [HbT] (due to pooling of highly absorbing hemoglobin), whereas edema involves buildup of water and should cause very small or no change in absorption.

Given our modest scattering change and small absorption change, the most likely interpretation of the scattering change is that AMS is associated with a mild, reversible cerebral edema. This is consistent with recent MRI findings (Dubowitz et al. 2009; Hunt et al. 2013), although not all (Mairer et al. 2012).

In terms of magnitudes, there are no data available to directly relate a noninvasively measured *μ*_s_' change to a change in BWC in adult humans. However, using the invasive data from Xie and colleagues as a rough calibration – where a 1% change in BWC was associated with a 1.55 cm^−1^ increase in *μ*_s_′ – our ∆*μ*_s_′ finding would translate to a ~0.23% increase in BWC. While this is a small change, Xie et al. used an invasive preparation in a small animal, whereas we conducted measurements noninvasively in adult humans. Noninvasive NIRS measures on the head blend changes in the scalp with changes in the brain according to a sensitivity profile that decreases exponentially in depth (Strangman et al. 2013). Given this, our estimate of +0.23% ∆BWC likely underestimates cerebral edema in our subjects. Both animal models and human measurements have generally reported a 1.5–2.5% change in BWC associated with moderate to severe traumatic brain injury edema (Marmarou et al. 2000; Cheng et al. 2010). Models of mild TBI have demonstrated more modest or nonsignificant increases in BWC (Kane et al. 2012). The estimated lower bound of +0.23% ∆BWC thus appears appropriately intermediate, and consistent with the modest and variable evidence of cerebral edema from prior cerebral imaging studies with AMS. This small effect is also consistent with the large effect found in high‐altitude cerebral edema (HACE), for which ample evidence of cerebral edema has been reported (Hackett et al. 1998; Gallagher and Hackett 2004).

These results support the prior hypothesis that AMS is associated with mildly elevated cerebral edema. The independence of AMS‐related scattering changes from changes associated with the environmental exposures further supports the notion that hypoxia induces modest cerebral edema, and AMS induces further edema. Near‐infrared spectroscopy measurements cannot directly determine whether edema is vasogenic versus cytotoxic, and so, cannot directly address the hypothesis that simple exposure to hypoxia may result in asymptomatic vasogenic edema, whereas AMS is specifically related to cytotoxic edema (Kallenberg et al. 2007; Schoonman et al. 2008). However, we did find AMS+ to be associated with significantly elevated scattering changes independent of NH exposure. This provides indirect evidence that hypoxia and AMS have distinct influences on cerebral edema. The results also suggest that having AMS is an additive effect over and above NH exposure, with a magnitude two‐thirds that of NH exposure alone. Though AMS was not an additive effect with respect to HH, this was the result of AMS+ being notably collinear with HH. Scattering did not appear to be significantly elevated with longer exercise duration, nor was duration of exposure a significant predictor of *μ*_s_′. Given the small effect sizes, however, one cannot completely rule out effects of exercise or exposure duration based on our negative findings.

Acute mountain sickness and HACE are often described as being on a pathophysiologic continuum, with common and benign AMS on one end, and rare, but potentially lethal HACE on the other. While it has not been compellingly demonstrated that AMS is indeed a “milder” form of HACE, our ONSD findings further support the role of increased ICP in development of AMS. Both ONSD and cerebral edema are known to correlate with elevated ICP, and significantly elevated ICP is a generally accepted late effect of HACE. The observed increases in ONSD in subjects with AMS were small, but are further substantiated by the significant ONSD increases associated with hypoxic environments and exercise. The [HbT] increases are consistent with the increase in ONSD, although the small magnitude of the [HbT] increases plus the potential crosstalk from the scattering changes limit the strength of any such inference (Strapazzon et al. 2014).

We had hypothesized that a substantially elevated ICP might suppress the hemodynamic response to Valsalva maneuvers and enhance the response to Mueller maneuvers. Individual Valsalva maneuvers generated reliable increases in oxy‐, deoxy‐ and total hemoglobin, and Mueller maneuvers generated reliable decreases in the same species. However, we found no evidence that hypoxic exposure or AMS affected these hemodynamic responses. We propose that this is because our maneuvers involved generating pressures of 40 mmHg, which overwhelmed any modest ICP elevation associated with AMS and/or hypoxia.

No significant differences between NH and HH were identified in any of our cerebral outcome variables. This would suggest that at least the cerebral responses to NH and HH were similar, presumably owing to the comparable reduced‐oxygen environments in these two conditions. This contrasts with prior studies showing NH and HH differ in terms of responses they produce in AMS symptoms (Roach et al. 1996), fluid balance (Loeppky et al. 2005), and ventilation (Tucker et al. 1983). Given the modest effect sizes between sea level (NN) and both 4400 m equivalent exposures, however, it is also possible that we failed to see significant differences between more subtle exposure differences (like those between NH and HH) due to the sensitivity of RF‐NIRS.

## Limitations

This study provides some of the first data regarding cerebral changes *during* the first 8 h of NH and HH exposures and when AMS symptoms were emerging. However, the three time points during exposure were not sufficient to investigate lead/lag relationships between variables. Also, the subjects that developed AMS+ within the 8 h exposures may be more susceptible to AMS, and hence their cerebral responses may not represent the larger population. Additionally, further work to calibrate the RF‐NIRS method against other neuroimaging techniques could enable more direct quantification of cerebral edema noninvasively. Nevertheless, this is the first study to observe significant increases in tissue scattering in subjects with AMS versus those without, consistent with mildly increased cerebral edema in AMS+.

## Conclusion

Our NIRS results support the hypothesis that even in asymptomatic individuals, exposure to either NH or HH results in measurable cerebral edema, and that AMS contributes additional cerebral edema. As compared with asymptomatic subjects, those with AMS also had evidence of increased cerebral perfusion and mildly elevated ICP, suggesting a cerebral response to AMS development. The nature, cause, time course, and interindividual variability of the additional edema associated with AMS, and associated perfusion or ICP changes remains to be understood.

## Conflict of Interest

The authors have no conflicts of interest to declare.
